# Comparison among cognitive diagnostic models for the TIMSS 2007 fourth grade mathematics assessment

**DOI:** 10.1371/journal.pone.0188691

**Published:** 2018-02-02

**Authors:** Kazuhiro Yamaguchi, Kensuke Okada

**Affiliations:** 1 Graduate School of Education, the University of Tokyo, Tokyo, Japan; 2 Department of Psychology, Senshu University, Kanagawa, Japan; Kyoto University, JAPAN

## Abstract

A variety of cognitive diagnostic models (CDMs) have been developed in recent years to help with the diagnostic assessment and evaluation of students. Each model makes different assumptions about the relationship between students’ achievement and skills, which makes it important to empirically investigate which CDMs better fit the actual data. In this study, we examined this question by comparatively fitting representative CDMs to the Trends in International Mathematics and Science Study (TIMSS) 2007 assessment data across seven countries. The following two major findings emerged. First, in accordance with former studies, CDMs had a better fit than did the item response theory models. Second, main effects models generally had a better fit than other parsimonious or the saturated models. Related to the second finding, the fit of the traditional parsimonious models such as the DINA and DINO models were not optimal. The empirical educational implications of these findings are discussed.

## Introduction

Assessing students’ current level is the first step towards improving their academic skills. Indeed, effective educational assessment is important because it helps inform students of the extent of their current knowledge, and can facilitate timely follow-up and support from teachers or parents [1]. One of the most familiar types of formal assessment is the achievement test, which measures what a student already knows or can do. In order to extract useful information from these achievement tests, a number of psychometric models have been developed.

One of the most famous and important set of models related to educational assessment, which are often used specifically for high-stakes tests, is item response theory (IRT) [2]. Among these models, the 1–3 parameter logistic (1–3 PL) models [3] are rather popular. However, because IRT models are not designed to model diagnostic information, they typically restrict examinees’ latent ability to be unidimensional or, at most, a few dimensions. Thus, IRT models might not be appropriate for modeling numerous attributes, which is often needed for educational diagnosis.

To overcome this issue, in the past few decades researchers have developed a new class of test models called the cognitive diagnostic models (CDMs) [4]. CDMs are probabilistic, confirmatory multidimensional latent variable models that typically have a complex loading structure [5]. From a broader perspective, CDMs can be regarded as a special case of the latent class model [6,7]. The key feature of CDMs is that they allow for multiple criterion-referenced interpretations and associated feedback for diagnostic purposes [5]. Unlike IRT models, CDMs can consider a variety of cognitive abilities or skills—called attributes—in solving the test items. The diagnostic information yielded from these models would in turn help students realize what they must study next, which can help them save time and resources. This is supported by Yeany and Miller’s meta-analysis [8], which showed that diagnostic information improves student learning effectiveness. Additionally, Tatsuoka and Tatsuoka [9] found that diagnostic feedback in remedial courses helped improve students’ mathematical ability. Furthermore, not only students but also teachers benefit from CDMs: teachers can review their curriculums based on concrete evidence, and can concentrate on teaching specific attributes that many of their students do not understand. Thus, CDMs have the potential to be a powerful basis for tailored education.

To be able to consider multiple attributes, CDMs use the Q-matrix, which represents the relations between the test items and attributes [10]. Table 1 shows a simple artificial example of a Q-matrix with three arithmetic attributes: addition/subtraction, multiplication, and division. For instance, in this Q-matrix, the item “103 + 203” requires only the addition/subtraction attribute for correct answering, whereas item “12 × 13” requires only the multiplication attribute. However, the item “21 ÷ 7–8 × 4” is different from the others because it requires all three attributes. The relationship shown in Q-matrixes is constructed through a literature review, expert discussion, and protocol analysis during the test [11].

**Table 1:** Example of a Q-matrix.

	Attributes		
Items	Addtion/Subtraction	Multiplication	Dividion
103 + 203	1	0	0
12 × 13	0	1	0
21 ÷ 7–8 × 4	1	1	1

In order to effectively apply CDMs, the item response function must be determined. Broadly speaking, CDMs comprise three major classes of models that differ in their item response functions: simple parsimonious models, main effects models, and the saturated model. The parsimonious models include the well-known deterministic-input noisy “and” gate (DINA) [12] and deterministic-input noisy “or” gate (DINO) [13] models, which have the longest history in the literature of CDMs. The main effect models assume that each attribute has an additive effect on the linear predictor without any interactions [14]. On the other hand, the saturated model includes all possible interactions between attributes in addition to the main effects [14]. A more detailed account of each type of model is given in the Cognitive Diagnostic Models section of this paper.

Because a variety of CDMs have been proposed, an important research question is which model better describes existing large-scale assessments. A number of past studies have compared several CDMs for large-scale educational assessments such as the Programme for International Student Assessment (PISA) and the Michigan English Language Assessment Battery (MELAB) reading test. Chen and de la Torre [15] found that the full generalized deterministic-input noisy and gate model (G-DINA model) [14] was comparatively the best model among the sub-models of the G-DINA framework for the reading section of the PISA. Li, Hunter, and Lei [16] compared the sub-models of the G-DINA for the MELAB reading test, and found that the full G-DINA model was a better fit in terms of its Akaike information criterion (AIC), whereas the additive CDM (A-CDM) [14], one of the main effects models, was superior in terms of the Bayesian information criterion (BIC). Jang [17] examined reading skills in English as a second language and applied the Fusion model [18], which is a kind of main effects CDM. Furthermore, Suzuki, Toyota, Yamaguchi, and Sun [19] applied the G-DINA models to the Kyoukenshiki Standardized Achievement Test, a norm-referenced mathematics test, when it was taken by first-year students of a Japanese junior high school; they found that the A-CDM fitted the data well. Selecting well-fitting model out of the various existing CDMs would facilitate, for example, international comparison of large-scale educational assessment [20, 21].

The Trends in Mathematics and Science Study (TIMSS) is an international assessment of the trends in mathematics and science achievements, which is run by the International Association for the Evaluation of Educational Achievement (IEA) [22]. The goal of the TIMSS is to provide information about students’ achievement in order to improve their learning [22]. Based on the discussion so far, it would be meaningful to apply CDMs to TIMSS data because this would help capture students’ current cognitive status as well as the direction of their learning. While the TIMSS was originally developed for a unidimensional scale in the IRT framework, CDMs might provide the additional benefits of individualized diagnosis and treatment. In support of this view, many studies have applied one of the CDMs to the TIMSS datasets [23–25]. In particular, Birenbaum, Tatsuoka, and Xin [23] pointed out the shortcomings of the IRT score-based feedback of the TIMSS, as this precludes an accurate diagnosis at the individual level. They also discussed the potential of a diagnostic approach in overcoming these challenges.

However, compared to other large-scale international assessments such as the PISA, there have been few comparative studies of CDMs for the TIMSS. In existing CDM studies of the TIMSS, the quantitative analysis was typically pre-determined by the researchers. For instance, several former studies [26–29] employed the rule space method (RSM) [30], a statistical pattern recognition method based on item response data. However, the RSM is not a probabilistic model, and does not employ likelihood-based estimation. Therefore, it is difficult to evaluate the magnitude of the errors in a probabilistic sense or to compare between different models based on information criteria using this method.

Another study that fitted CDMs to large-scale educational data was by Chen and de la Torre [15], who considered the G-DINA family of models for data from the reading section of the PISA. Both the TIMSS and PISA are large-scale international educational assessments that originally aimed to compare achievements among countries in the framework of the unidimensional IRT model. Thus, in the same manner as Chen and de la Torre [15], it would be meaningful to apply CDMs to TIMSS data in order to extract diagnostic information.

To the best of our knowledge, there are three existing TIMSS studies that have applied probabilistic models and likelihood-based inference. First, Lee, Park, and Taylan [31] compared the deterministic-input, noisy and gate model (DINA model) [30], a recently developed latent-class-based stochastic CDM, and IRT models for the TIMSS fourth grade mathematics assessment in a U.S. national sample and samples from only Minnesota and Massachusetts. They found that the DINA model fit better than did the IRT models in terms of both AIC and BIC. Second, Choi, Lee, and Park [32] compared Korean and U.S. national samples using the DINA model for the TIMSS 2003 eighth grade mathematics assessment. They did not conduct a formal likelihood-based model comparison, and instead examined the differences in attribute mastering profile between the two countries. Finally, Yamaguchi and Okada [33] conducted a model comparison study using only Japanese data, but their domestic study was not comprehensive, serving only as preliminary results.

These existing studies have provided a novel viewpoint for modeling the TIMSS assessment data. However, the following main two questions remain. First, Lee et al.’s [31] finding that the CDM was a better fit to the data than the IRT model was based only on U.S. samples. Therefore, it seems reasonable to ask whether this finding can be generalized to other countries outside of the United States or not, as one would expect that samples within a single country are relatively homogeneous (i.e. the United States). Thus, evaluation of the generalizability of these findings seems necessary.

Second, Lee et al. [31] focused solely on the DINA model; no other CDMs were considered, despite the fact that numerous others exist (see the Cognitive Diagnostic Models section below). As we stated previously, CDMs can be divided into three different classes—simple parsimonious models, main effects models, and the saturated model—in terms of their representation of item response behavior. Thus, it seems pertinent to question which class of CDMs is a better fit to the TIMSS.

Related to the second question, if CDMs do in fact fit the data better than do IRT models, which specific CDMs are more representative of the characteristics of the TIMSS data? Although the good model might differ between countries, we may be able to identify trends that reflect a difference in culture or students’ cognitive characteristics. Originally, the TIMSS was not designed for diagnostic purposes. However, it would still be meaningful to investigate what type of CDMs achieve a good fit to the current TIMSS items. CDMs can tell us what abilities students require to solve the items and which abilities students have gained because the attributes of the test would be selected by a sophisticated search and because each item response function utilizes different assumptions for solving items; such information might not be obtained from IRT models. In other words, well-fitted CDMs would provide us with information on the individual differences in skills that students would need for solving the test.

The main objective of the current study was to attempt to answer the above two questions by analyzing the TIMSS 2007 assessment data in seven countries. First, we sought to replicate the previous finding (Lee et al. [31]) that CDM models are a better fit than IRT models in countries other than the United States. This would tell us whether Lee et al.’s findings were unique to the United States or whether they can be generalized to other countries. Second, we exploratorily investigated whether parsimonious models, main effect models, or the saturated model better fit the TIMSS 2007 assessment. This was done by conducting a statistical model comparison among the CDMs using probabilistic models and likelihood-based estimation. In addition, we sought to determine the best-fitting models for each of seven countries. This was achieved using the same model comparison and estimation, with a focus on each specific country rather than the aggregate. By investigating which type of model better fits to TIMSS data, we might, for example, obtain new insights that could help in the future diagnostic extension of the TIMSS.

## Methods

### Data

Following Lee et al. [31], we analyzed the assessment data from booklets four and five of the TIMSS 2007 fourth grade mathematics assessment. We analyzed the data of not only the United States, which was already analyzed in Lee et al. [31], but also in six other countries. These countries were chosen because of their places in the mathematics achievement ranking depicted in Exhibit 1.1 in Martin et al. [22]. Specifically, we chose two countries each from among the high-, average-, and low-ranked countries, including Hong Kong SAR and Singapore from the high-ranked countries; Slovenia and Armenia from the average-ranked countries; and Qatar and Yemen from the lower-ranked countries. Note that the IEA uses “countries” in all cases to differentiate between participating entities. We use the same terminology throughout this paper, even though Hong Kong is a Special Administrative Region (SAR) of China. We excluded the data of examinees who had no or only one response to the items because we would not be able to estimate these participants’ attribute mastery patterns. Table 2 depicts each country’s sample size by gender. The largest sample size was 1,130 (in the U.S.) and the smallest was 543 in Hong Kong SAR.

**Table 2:** Sample size in each country.

Country	Girls	Boys	Sample size
USA	587	543	1130
Hong Kong SAR	252	291	543
Singapore	345	372	717
Slovenia	319	301	620
Armenia	287	299	586
Qatar	519	480	999
Yemen	375	461	836

### Attributes and Q-matrix

By design, the TIMSS 2007 mathematics achievement test has three content domains, each of which has multiple topic areas. Each topic area comprises 38 objectives. Lee et al. [31] asked three researchers with degrees in mathematics education and two domain expert researchers to determine the preliminary attributes required for the data from the topic area. Then, Lee et al. [31] modified these attributes to better suit the research objective, ultimately generating a total of fifteen attributes. We employed the same attributes and Q-matrix; the complete Q-matrix used in this study is depicted in Table 3 of Lee et al. [31]. The attributes formed three content domains [22]: number (NUM), geometric shapes and measurement (GM), and data display (DD). Each domain comprised several attribute areas, each of which in turn comprised several specific attributes.

**Table 3:** Mean (SD) rate that each attribute is required for correctly answering items.

Attributes in Number (NUM) domain			Attributes in Geometric Shapes & Measurement (GM) domain			Attributes in Data & Display (DD) domain		
Attribute number	Mean	(SD)	Attribute number	Mean	(SD)	Attribute number	Mean	(SD)
1	.240	(.427)	9	.120	(.325)	13	.160	(.367)
2	.640	(.480)	10	.280	(.449)	14	.120	(.325)
3	.440	(.496)	11	.080	(.271)	15	.080	(.271)
4	.120	(.325)	12	.120	(.325)			
5	.120	(.325)						
6	.080	(.271)						
7	.080	(.271)						
8	.120	(.325)						

Note. The data comprise 25 items (Booklets 4 and 5).

The NUM domain comprised four attribute areas: “whole numbers,” “fractions & decimals,” “number sentences with whole numbers,” and “patterns & relationships.” There are a total of eight attributes in the NUM domain, such as “1. Representing, comparing, and ordering whole numbers as well as demonstrating knowledge of place value” and “2. Recognize multiples, computing with whole numbers using the four operations, and estimating computations.” The GM domain comprises three attribute areas: “lines & angles,” “two- and three-dimensional shapes,” and “location & movement.” There are a total of four attributes in the GM domain, such as “9. Measure, estimate, and understand properties of lines and angles and be able to draw them” and “10. Classify, compare, and recognize geometric figures and shapes and their relationships and elementary properties.” Finally, the DD domain, which is the smallest of the three, has only three attributes, such as “13. Read data from tables, pictographs, bar graphs, and pie charts” and “15. Understanding different representations and organizing data using tables, pictographs, and bar graphs”.

The means and standard deviations of the rates that each attribute is required for correctly answering items are shown in Table 3. Items require on average 2.80 attributes (*SD* = 1.20, Min = 1, Max = 6). Almost all of the items required more than one attribute to be correctly answered. Thus, the Q-matrix has a complicated structure.

### Cognitive diagnostic models

We considered and compared the wide range of CDMs that make up the sub-models of the G-DINA model framework [14]. Specifically, we considered two parsimonious models (the DINA [12] and DINO [13] models), three main effects models (A-CDM [14], linear logistic model (LLM) [34], and reduced reparametrized unified model (R-RUM) [18]), and the saturated full G-DINA model. The models are summarized in Table 4. In the following paragraphs, we provide a brief overview of the G-DINA family of models in order to clarify the models that we are considering. For a more detailed explanation of the G-DINA model framework, including discussions on model identification and parameter constraints, please see de la Torre [14].

**Table 4:** Summary of sub-models of G-DINA model framework.

Model	Link function	Model type	Main effects	Interaction effects	#Item parameters
G-DINA	Identity	Saturated	✓	✓	$2^{K_{j}^{*}}$
DINA	Identity	Parsimonious		✓	2
DINO	Identity	Parsimonious	✓	✓	2
A-CDM	Identity	Main effects	✓		${1+K}_{j}^{*}$
LLM	logit	Main effects	✓		${1+K}_{j}^{*}$
R-RUM	log	Main effects	✓		${1+K}_{j}^{*}$

Note. The check mark, ✓, indicates that we needed to estimate the terms.

Much like the analysis of variance (ANOVA) model, the G-DINA model framework comprises both main effect and interaction terms. In other words, the model considers both the unique effects of the attributes as well as the combined effects of more than two attributes at once. Let us denote the *l*th attribute mastery pattern for item *j* as $\alpha_{lj}^{*}\;(l=1,\ldots ,K_{j}^{*})$, which is a vector comprising 1s for the mastered attribute and 0s for the attribute that is not yet mastered. For example, consider three attributes and the attribute mastery pattern vector $\alpha_{lj}^{*}=[0,1,0]$; this attribute mastery pattern indicates that the first and the third attributes have not been mastered while the second attribute has been mastered. $K_{j}^{*}$ indicates the number of attributes required for item *j*, which is always less than the total number of attributes *K*. Then, in the G-DINA model framework, the probability of a correct item response (*X*_*j*_ = 1) for participants who have the attribute mastery pattern vector $\alpha_{lj}^{*}$ for item *j*, is given as follows:

$$P_{j}(\alpha_{lj}^{*})=\mathrm{Pr}\left(X_{j}=1|\alpha_{lj}^{*}\right)=\delta_{j0}+\sum_{k=1}^{K_{j}^{*}}\delta_{jk}\alpha_{lk}+\sum_{k^{\prime }=k+1}^{K_{j}^{*}}\sum_{k=1}^{K_{j}^{*}-1}\delta_{jkk^{\prime }}\alpha_{lk}\alpha_{lk^{\prime }}+\cdots +\delta_{j12∙∙∙K_{j}^{*}}\prod_{k=1}^{K_{j}^{*}}\alpha_{lk}.$$ (1)

Here, δ_*j*0_ is an intercept term representing the correct response probability when an examinee has not mastered an attribute needed for the item. Main effect term δ_*jk*_ represents the increment of correct response probability when an examinee has the *k*th attribute α_*k*_. First order interaction term $\delta_{jkk^{\prime }},k\neq k^{\prime },$ represents the amount of change in the correct response probability when an examinee has attributes α_*k*_ and $\alpha_{k^{\prime }}$. In a similar fashion, $\delta_{j12∙∙∙K_{j}^{*}}$ represents the highest order interaction for item *j*.

By constraining some of the components of Eq (1) to zero, reparametrizing some of the components, and suitably choosing the link function, we can obtain the various sub-models of the G-DINA model framework. The DINA and DINO models are the most constrained of the G-DINA sub-models. The DINA model contains only an intercept parameter and the highest interaction term. The DINO model also has only two parameters for each item, and has somewhat complicated constraints for the main effect and interaction terms.

The A-CDM, LLM, and R-RUM have only the intercept and main effects parameters, but no interaction parameters. These models are less constrained than are the DINA and DINO models. The difference in these model is in their link function. The A-CDM model has an identity link function, the LLM model has a logit function, and the R-RUM has a log-link function; thus, the interpretation of the parameters differs from each other to some degree. However, these three models are similar in terms of their parameterization.

As mentioned above, the saturated full G-DINA model has not only the intercept and main effects parameters, but also the interaction parameters between all possible combinations of attributes. This means that the saturated full G-DINA model is the most fully parameterized model in the framework.

Note that there are other ways of classifying the CDMs, such as the compensatory and noncompensatory family of models [35]. However, we chose to compare between the above three classes of models because this classification reflects the complexity of the models. Thus, the comparative results may be suggestive in understanding the required complexity of the models for the given problem.

### Data analysis

We analyzed each country’s data using the full G-DINA model and its sub-models as well as the 1–3PL IRT models. Specifically, we compared the fit of three IRT models and six CDMs for each country by computing the deviance (− 2 log(Likelihood), and then computing the Akaike information criterion (AIC; Deviance + 2 × [number of item parameters]) and Bayesian information criterion (BIC; Deviance + [number of item parameters] × log[sample size]). We used the AIC and BIC for model comparison.

For completeness, we also calculated two absolute fit measures: the mean absolute deviation correlation (MADcor) [35] and standardized root mean square residual (SRMSR) [36]. It is expected that IRT models would provide a better absolute fit than would CDMs because while IRT models assume only continuous latent variables, CDMs assume discrete latent variables that correspond to attribute mastery/nonmastery. It is also expected that CDMs with a greater number of parameters would have a better absolute fit than would those with fewer parameters. Therefore, our primary measures of model comparison were the AIC and BIC, and the absolute measures should only be considered as a reference. The MADcor is defined as

$$MADcor=\frac{2}{J(J-1)}\sum_{j^{\prime }<j}\left|r_{j^{\prime }j}-{\hat{r}}_{j^{\prime }j}\right|,$$ (2)

where $r_{j^{\prime }j}$ is the sample observed correlation coefficient between items *j*′ and *j*, and ${\hat{r}}_{j^{\prime }j}$ is the expected correlation coefficient. Thus, the MADcor corresponds to the mean of the absolute difference between ${\hat{r}}_{j^{\prime }j}$ and $r_{j^{\prime }j}$ for all item pairs. Likewise, the SRMSR is defined as

$$SRMSR=\sqrt{\frac{2}{J(J-1)}\sum_{j^{\prime }<j}{\left(r_{j^{\prime }j}-{\hat{r}}_{j^{\prime }j}\right)}^{2}}.$$ (3)

The SRMSR corresponds to the root mean squared difference between the expected and observed correlations of all item pairs. These formulae can be found in the help files of the CDM package [37].

For the CDM, we employed the GDINA package [38] for the open-source statistical language R. Marginal maximum likelihood estimation (MMLE) was used to estimate the item parameters and participants’ attribute mastery statuses. We used twelve starting values and selected the highest log-likelihood solution to avoid local solutions. For the IRT models, we employed the tpm function in the ltm package [39]. The MMLE was used for estimating the item parameters, while empirical Bayes estimation was used to estimate the latent traits of the examinees. To calculate the absolute fit indices in the IRT models, we used the tam.modelfit function in the TAM package [40] based on item parameters estimated with tpm function.

## Results

### Model comparison

The results of the model comparison are shown in Table 5. The best model for each criterion within each country is shaded. Slovenia, Qatar, and Yemen exhibited improper solutions under the 3PL IRT model, but we have presented the values for reference in Table 5 in any case. Because the full G-DINA model was a saturated model, it had the smallest deviance in all of the countries. For the MADcor and SRMSR, we used complete pairs to calculate the correlations. Note that in Qatar, all responses were zero for one item (M031247), and thus this item was removed from the calculation.

**Table 5:** Comparison of IRT models and CDMs in each country.

Country	IRT/CDM	Model	Deviance	AIC	BIC	MADcor	SRMSR	#Item Parameters
USA	IRT	3PL	21197.05	21347.05	21724.29	.030	.041	75
		2PL	21245.88	21345.88	21597.38	.031	.042	50
		1PL	21605.52	21655.52	21781.27	.061	.078	25
	CDM	G-DINA	18383.19	18907.19	20225.04	.072	.072	262
		DINA	21173.55	21273.55	21525.05	.084	.077	50
		DINO	21244.69	21344.69	21596.19	.105	.102	50
		A-CDM	18649.78	18839.78	19317.62	.081	.082	95
		LLM	19088.42	19278.42	19756.27	.070	.074	95
		R-RUM	18584.39	18774.39	19252.23	.080	.081	95
Hong Kong SAR	IRT	3PL	7812.17	7962.17	8284.45	.045	.059	75
		2PL	7841.21	7941.21	8156.06	.046	.059	50
		1PL	7996.71	8046.71	8154.14	.076	.094	25
	CDM	G-DINA	6437.60	6961.60	8087.44	.091	.089	262
		DINA	7707.45	7807.45	8022.31	.135	.121	50
		DINO	7712.32	7812.32	8027.18	.130	.115	50
		A-CDM	6653.40	6843.40	7251.63	.101	.096	95
		LLM	6582.56	6772.56	7180.79	.095	.093	95
		R-RUM	6783.54	6973.54	7381.77	.115	.107	95
Singapore	IRT	3PL	10554.09	10704.09	11047.22	.035	.047	75
		2PL	10600.62	10700.62	10929.37	.038	.050	50
		1PL	10976.23	11026.23	11140.60	.092	.114	25
	CDM	GDINA	8926.62	9450.62	10649.29	.103	.101	262
		DINA	10561.27	10661.27	10890.03	.155	.136	50
		DINO	10645.89	10745.89	10974.64	.169	.155	50
		A-CDM	9261.37	9451.37	9886.00	.108	.105	95
		LLM	9406.80	9596.80	10031.44	.115	.111	95
		R-RUM	9308.16	9498.16	9932.79	.111	.106	95
Slovenia	IRT	3PL	10697.15	10847.15	11179.38	.038	.049	75
		2PL	10725.67	10825.67	11047.15	.040	.052	50
		1PL	10908.81	10958.81	11069.55	.065	.085	25
	CDM	G-DINA	9178.72	9702.72	10863.31	.080	.079	262
		DINA	10552.44	10652.44	10873.92	.123	.113	50
		DINO	10622.81	10722.81	10944.29	.124	.114	50
		A-CDM	9436.26	9626.26	10047.08	.088	.083	95
		LLM	9388.83	9578.83	9999.65	.091	.091	95
		R-RUM	9234.20	9424.20	9845.02	.095	.097	95
Armenia	IRT	3PL	9420.97	9570.97	9899.10	.060	.082	75
		2PL	9439.23	9539.23	9757.98	.060	.083	50
		1PL	9564.28	9614.28	9723.66	.082	.106	25
	CDM	G-DINA	7795.31	8319.31	9465.12	.116	.111	262
		DINA	9256.33	9356.33	9575.00	.146	.136	50
		DINO	9217.19	9317.19	9535.86	.153	.140	50
		A-CDM	8063.70	8253.70	8669.16	.121	.120	95
		LLM	8069.33	8259.33	8674.80	.120	.119	95
		R-RUM	8173.22	8363.22	8778.68	.123	.119	95
Qatar	IRT	3PL	13112.33	13262.33	13630.41	.044	.057	75
		2PL	13188.73	13288.73	13534.12	.047	.059	50
		1PL	13564.05	13614.05	13736.74	.075	.091	25
	CDM	G-DINA	11479.83	12003.83	13289.40	.093	.094	262
		DINA	13045.53	13145.53	13390.87	.098	.089	50
		DINO	13008.34	13108.34	13353.68	.094	.087	50
		A-CDM	11820.48	12010.48	12476.63	.092	.097	95
		LLM	11720.40	11910.40	12376.54	.101	.104	95
		R-RUM	11949.82	12139.82	12605.96	.100	.105	95
Yemen	IRT	3PL	10402.46	10552.46	10907.20	.056	.073	75
		2PL	10448.07	10548.07	10784.56	.058	.077	50
		1PL	10886.10	10936.10	11054.34	.098	.125	25
	CDM	G-DINA	8978.58	9502.58	10741.48	.125	.137	262
		DINA	10313.60	10413.60	10650.03	.129	.124	50
		DINO	10295.34	10395.34	10631.77	.148	.143	50
		A-CDM	9291.09	9481.09	9930.31	.141	.153	95
		LLM	9247.31	9437.31	9886.53	.140	.151	95
		R-RUM	9314.84	9504.84	9954.06	.134	.145	95

Note. CDM = cognitive diagnostic model; IRT = item response theory; 1–3PL = 1–3 parameter logistic model. The best value for each criterion within each country is shaded.

According to the AIC and BIC, the same model was chosen in almost all countries, with the exception of Singapore. Moreover, the CDMs showed a better fit than did the 1–3PL IRT models in all of the countries. Thus, this replicates the previous finding by Lee et al. [31] that the CDMs show a better fit than the IRT models. More specifically, for the United States and Slovenia, the main effect model R-RUM was the best fitting model. In the five other countries, the other main effect models were selected as the best fitting models: the LLM was selected in Hong Kong SAR, Qatar, and Yemen, while the A-CDM was selected in Singapore and Armenia. The R-RUM, LLM, and A-CDM are all the main effects model. Therefore, the main effects models were selected in almost all countries from the perspective of the information criteria.

As theoretically expected, absolute fit measures were minimal in the 3PL model in all countries. Among the CDMs, the saturated G-DINA model achieved the smallest absolute fit measures.

Using the best-fitting model based on the BIC for each country, we provide the summary statistics of the estimated number of mastered attributes in each country in Table 6. We chose the BIC rather than the AIC because it has greater penalties for overfitting [41]. This result is based on expected a posteriori estimation; in other words, participants were treated as having mastered the attribute when their posterior mean mastery probability exceeded .5. In Hong Kong SAR and Singapore, examinees had mastered six attributes of NUM, three attributes of GM, and two or three attributes of DD. Thus, examinees lacked only one or two attributes for each content domain in these two countries. In Slovenia and Armenia, examinees had mastered fewer attributes than did those in Honk Kong SAR and Singapore, but more than those in Qatar and Yemen. The results for Slovenia and Armenia were in fact similar to those in the United States. Qatar and Yemen had only one or two attributes mastered in each area on average. Furthermore, based on the estimated medians, it was postulated that most students had mastered only one or none of the attributes in each area. Taken together, these results indicate that the attribute mastery patterns differed noticeably but in an interpretable manner among the countries, and that the estimated mastered attributes had a corresponding relationship with mathematical competency.

**Table 6:** Summary statistics of the number of mastered attributes.

Country	Attribute	Mean	(SD)	Median	Skewness	Kurtosis
USA	NUM	4.263	(2.270)	4	0.082	-1.009
	GM	1.826	(1.115)	2	0.251	-0.687
	DD	2.174	(0.865)	2	-0.737	-0.351
	All	8.263	(3.498)	8	-0.027	-0.939
Hong Kong SAR	NUM	5.722	(1.846)	6	-0.652	-0.160
	GM	2.972	(1.002)	3	-0.702	-0.315
	DD	2.230	(0.868)	2	-0.951	0.131
	All	10.924	(2.999)	11	-0.539	-0.289
Singapore	NUM	5.922	(2.154)	7	-0.948	-0.021
	GM	2.580	(1.235)	3	-0.444	-0.891
	DD	2.379	(0.867)	3	-1.197	0.379
	All	10.881	(3.812)	12	-0.922	-0.114
Slovenia	NUM	4.590	(2.222)	5	-0.278	-0.926
	GM	2.174	(1.075)	2	-0.178	-0.532
	DD	1.773	(1.010)	2	-0.203	-1.137
	All	8.537	(3.527)	9	-0.256	-0.643
Armenia	NUM	4.790	(2.039)	5	-0.317	-0.948
	GM	2.348	(0.983)	2	-0.124	-0.475
	DD	1.539	(1.073)	1	-0.002	-1.262
	All	8.677	(3.404)	9	-0.236	-0.862
Qatar	NUM	2.004	(1.208)	2	0.729	0.271
	GM	1.209	(1.007)	1	0.515	-0.391
	DD	1.019	(0.912)	1	0.532	-0.598
	All	4.232	(2.132)	4	0.596	0.137
Yemen	NUM	2.300	(1.320)	2	1.159	1.753
	GM	0.962	(0.919)	1	0.686	-0.300
	DD	0.610	(0.712)	0	0.884	0.060
	All	3.872	(2.142)	4	1.040	1.772

Note. The numbers were based on expected a posteriori estimation. NUM = Number, GM = Geometric Shapes & Measurement; DD = Data & Display; All = sum of three content domains.

### Correlations between IRT and CDMs scores

If the students’ achievement estimated from the CDM models do not correlate with that from the IRT models, it might indicate that these two models measure completely different aspects of student traits. We therefore conducted two additional analyses to evaluate the correlation between the attributes mastery patterns and the IRT-based unidimensional latent traits. We employed the best fitting model for each country to estimate students’ attribute mastery patterns. This was because, based on both the current and former studies, it would be too strict to assume that students’ item response functions would be the same across countries.

First, in each country, we calculated the correlations between the number of mastered attributes, which was estimated from the best-fitted CDM, and the proficiency, which was estimated from the 2PL IRT model. The results are shown in Table 7. For all attributes, we observed moderate to strong correlations in all countries. Qatar and Yemen had moderate correlations in all areas, especially DD. The United States, Hong Kong SAR, Singapore, Slovenia, and Armenia all had strong correlations in the NUM domain, and had correlations of more than .50 but less than .80 in GM and DD.

**Table 7:** Correlations between the unidimensional proficiency and the number of mastered attributes.

	NUM		GM		DD		All	
Country	*r*	95% CI	*r*	95% CI	*r*	95% CI	*r*	95% CI
USA	.878	[.864, .891]	.558	[.517, .597]	.517	[.636, .701]	.913	[.903, .922]
Hong Kong SAR	.850	[.825, .872]	.591	[.534, .643]	.534	[.588, .687]	.906	[.890, .920]
Singapore	.879	[.861, .895]	.751	[.717, .781]	.717	[.730, .791]	.913	[.900, .925]
Slovenia	.895	[.878, .910]	.516	[.455, .571]	.455	[.647, .729]	.919	[.905, .930]
Armenia	.853	[.829, .873]	.649	[.600, .694]	.600	[.618, .709]	.908	[.893, .921]
Qatar	.534	[.488, .577]	.402	[.349, .453]	.349	[.222, .336]	.612	[.572, .650]
Yemen	.441	[.385, .494]	.426	[.368, .480]	.368	[.166, .294]	.531	[.481, .578]

Note. NUM = Number; GM = Geometric Shapes & Measurement; DD = Data & Display; All = sum of three content domains. All correlations were significant (*p* < .001).

Second, we calculated the correlations between the average number of mastered attributes and the official TIMSS 2007 achievement score for each country (see Exhibit 1.1 in Martin et al. [22]). This result is shown in Table 8. The sample size here was equal to the number of countries (i.e., seven). Thus, the resultant confidence interval was rather wide. However, the obtained correlations were notably strong, with all point-estimates exceeding .90. This indicates that number of mastered attributes can be considered a good indicator of general mathematical ability. The results of these above two analyses can be interpreted as evidence of the validity of the attributes we considered in this study.

**Table 8:** Correlations between the official TIMSS 2007 achievement scores and the average number of mastered attributes.

	*r*	*p*	95% CI
NUM	.962	< .001	[.758, .995]
GM	.941	.002	[.645, .991]
DD	.969	< .001	[.800, .996]
All	.984	< .001	[.892, .998]

Note. *n* = 7 (number of countries). NUM = Number, GM = Geometric Shapes & Measurement, DD = Data & Display, All = sum of three content domains.

## Discussion

The findings of this current study for the TIMSS 2007 mathematical assessment can be summarized as follows in light of our two objectives. First, CDMs fitted better than did the IRT models in all of the countries considered. Second, the main effects models were found to fit better than were the parsimonious models or the saturated model in almost all of the countries. In other words, although we observed some variations between countries in terms of the best-fitted CDMs, the traditional, less-parameterized CDMs such as the DINA and DINO models were generally not selected as best-fitting models.

The first main finding is consistent with previous findings in the United States (Lee et al. [31]); thus, our study provides evidence of the generalizability of this claim. The fact that CDMs fit better than did the IRT models suggests that to succeed in the TIMSS mathematics assessment, students require more than one skill. While unidimensional IRT models might be helpful for ordering students on a single scale in high-stakes test, they are likely to be too simple to explain students’ cognitive process of problem solving. Thus, unlike IRT models, CDMs reflect actual students’ item response behavior based on the TIMSS objectives.

Of course, the choice between the CDMs and IRT models clearly depends on the objective of the analysis. For example, unidimensional IRT models might be more suitable when the objective is not to diagnose, but rather to order the examinees based on a specific unidimensional proficiency, such as when the aim is to develop large-scale computer-based adaptive testing. However, our finding that CDMs generally have better fit than the IRT models suggests that, when their application is suitable, CDMs can help us extract considerable information, including diagnostic information, about examinees.

Our second finding was that the main effects models were selected as the best-fitting models in almost all countries. Specifically, in most countries, the selected models were R-RUM, A-CDM, or LLM. This result suggests that the main effects models might better explain the structure of the TIMSS assessment as compared to the other CDMs. On the other hand, the DINA and DINO models generally had worse fits than did the main effects models, suggesting that these parsimonious models are too simple to apply. Thus, the current results suggest that the TIMSS 2007 fourth grade mathematics assessment might not require students to have all of the designated attributes for each item in order to correctly answer that item. In other words, it might be reasonable to think that students could separately, rather than simultaneously, apply their knowledge to solve the problems. Both the DINA and DINO models assume that respondents can be classified into two groups (being able to answer correctly or not) for each item. On the other hand, other CDMs examined in this study classify respondents into more than two groups, and thus these models can express group differences more flexibly than can the DINA and DINO model.

We should, however, be careful in interpreting this result because it is based on the assumption that all items have the same item response function within a country. The assumption could be too rigid; each item might have different item response functions in real situations. Still, this is one of the common assumptions of CDMs, and we believe that the result could provide a hint in making use of the TIMSS data for diagnostic purposes. For example, the current TIMSS test items could require multiple cognitive skills to solve, and thus it might be better to develop items that simultaneously involve various cognitive skills for DINA-model-based diagnostic tests.

In this study, we chose a wide range of countries in terms of TIMSS achievement score. The trend in the selected models for the TIMSS data might not relate to the TIMSS official score, which reflects mathematics ability—rather, model selection could depend on the sample. Still, results showed that the models selected in each country were largely main effects models, which were generally better than were other, more restricted or complicated models. CDMs consider only individual cognitive situations, but not individual environmental information. The country differences might therefore derive from differences in the education system or curricula between the countries. TIMSS data are rich in information about students’ learning customs, school curricula, and parents' information, and so on. Therefore, in the future, it would help to examine the relationship between diagnostic results and students’ background data using TIMSS data.

The finding that the DINA and DINO models achieved worse fit than did the other, more relaxed CDMs might be important for model developers. Traditionally, model development studies have used the DINA model as their basis because it is one of the first probabilistic CDMs to have been developed. By showing that the DINA model might actually be too restrictive to reflect actual students’ knowledge status, future model development studies might give further weight to the empirical fit of the models to a real dataset.

While these findings are all based on the TIMSS 2007 mathematics assessment, we believe that they might have implications beyond it. For example, in educational diagnostic assessment, quite a few studies (e.g., [31, 32, 42]) have applied the DINA model. For example, Sun, Suzuki, and Kakinuma [42] analyzed the fraction test for sixth grade students using the DINA model, and then gave them actual feedback based on the DINA model diagnosis. However, based on our results, the DINA model may be too restrictive for modeling item responses. Thus, more relaxed CDMs, such as the A-CDM, LLM, or R-RUM, might provide a better fit to this assessment. Of course, this claim must be tested; if it holds, future studies using the more relaxed CDMs might be able to more accurately estimate students’ knowledge, which would help in constructing more effective learning environments.

Of course, generally, the goodness-of-fit in CDMs depends not only the item response function but also the settings of the Q-matrix. In the current study, for our objective, we used the same Q-matrix as in a former study. We might be able to expect better model fit if we design a test for the DINA model using items that better fit the DINA.

In some former studies, there were conflicting results about whether the main effects models or the other models fit better to mathematics tests. For example, Roussos, Templin, and Henson [43] reported that “the assessment of mixed number subtraction included methods for model comparison, ending with the selection of the DINA model as the best fitting (p. 305).” This claim might be valid if the test was fully designed using the DINA model, but it does not imply that the DINA models are more appropriate for mathematics tests in general. Our results therefore present the possibility that if a mathematics test was not originally built for diagnostic purposes but fitted by CDMs, main effects models might better explain its structure.

We should also discuss the difference in the dimensionality of the CDM attributes and IRT’s proficiency parameter; in other words, we might interpret them differently. IRT’s unidimensional proficiency could in reality comprise a rich set of mathematical skills, knowledge, and understanding that are relevant to the test. Thus, the construct that is represented by the IRT’s proficiency parameter may actually be a composite of numerous mathematical components. On the other hand, the attributes of CDMs are included as categorical latent variables that correspond to somewhat narrow learning components with good grain size. The considerable difference between these parameter interpretation of these types of models might therefore render comparison between the models inappropriate. IRT models might be used for linking or equating items to make a common scale used for assessing multiple countries, even though they might not capture students’ actual test answering behaviors. CDMs, by contrast, might be used to better reflect students’ cognitive abilities.

Of course, this study has several limitations. In this study, we analyzed the results of only seven countries, despite the fact that a rather large number of countries participate in the TIMSS assessment. Future studies would evaluate the generalizability of our findings in other countries or other time points.

Furthermore, in this study, we employed the attributes and Q-matrix constructed by Lee et al. [31]. Using Lee et al.’s Q-matrix was most appropriate for our two objectives because it let us maintain comparability with previous studies. In other words, this choice of Q-matrix helped us replicate and extend their findings. On the other hand, the Q-matrix developed in Lee et al. [31] does not satisfy the identifiability condition. Recent studies [44–46] have found that when the Q-matrix is incomplete, the model parameters are unidentifiable. A Q-matrix is said to be complete when it contains items requiring only one attribute for each attribute; otherwise it is incomplete [46]. The Q-matrix used in this study was incomplete, which means that it cannot satisfy the identifiability condition.

Even so, this identifiability issue should not affect the maximized model likelihood. In the unidentified model, it is the parameter estimates with the highest likelihood value that are not uniquely determined. Even in such a case, the maximized likelihood value itself is still appropriate. Thus, even when parameters are not identified, we can perform model comparisons based on likelihood. Exploratory factor analysis model presents a good example. Specifically, an exploratory factor model has a parameter identification issue, that is, any rotation of the factor loadings does not change the maximized likelihood. Despite this, it is a very widely used model in educational and psychological science, allowing researchers to compare models with different numbers of factors based on their likelihood.

That being said, in the future study, it would be fruitful to further reduce the number of attributes used in the current study, leaving only the most stable, essential attributes. A former study on clarifying the structure of attributes might help in identifying such essential attributes [47].

Furthermore, in recent years, there have been a number of novel methodological developments in estimating the Q-matrix based on data. For example, de la Torre and Chiu [48] proposed a new discrimination index that can be used to empirically validate the Q-matrix by identifying and replacing its misspecified entries. Chen [49] proposed an alternative residual-based approach to validate Q-matrix specifications, while a Bayesian estimation method for estimating the Q-matrix of the DINA model was developed by Chen, Culpepper, Chen, and Douglas [50]. Although these methods are still being developed, and have not been extensively tested empirically, methodological advances might help us in better constructing and validating the application of CDMs. Thus, it would be ideal for future studies to re-investigate the Q-matrix specification in the TIMSS assessment by using these Q-matrix estimation methods.

CDMs have computational difficulties in their solutions, such as the local maxima. To tackle this issue in the present study, we employed multiple starting values. Another possible way to avoid this issue would be to use a different estimation algorithm, such as Bayesian or simulated annealing approaches.

Another future research topic includes the interpretation of the results in view of the microscopic item-scoring mechanism and the country-specific curricula. To actually apply diagnostic information, such a detailed investigation would be necessary. Discussion based on real curricula would enable greater utility for CDMs. It would also be interesting to investigate the relationships between the contents taught in each country and CDM results.
